# Microvascular Dysfunction as a Bridge between Chronic Obstructive Pulmonary Disease and Ischemic Events: Microvascular Dysfunction linking COPD and Ischemic

**DOI:** 10.31661/gmj.v14i.3937

**Published:** 2025-07-27

**Authors:** Ali Amiri, Roohollah Rahbani, Mohsen Abbasnezhad, Mehdi Maleki, Fatemeh Dadgar, Negar Jafari

**Affiliations:** ^1^ Pulmonary Subspecialty, Lorestan University of Medical Sciences, Khorramabad, Iran; ^2^ I.M. Sechenov First Moscow State Medical University, Moscow, Russia; ^3^ Cardiovascular Research Center, Tabriz University of Medical Sciences, Tabriz, Iran; ^4^ Department of Internal Medicine, Lorestan University of Medical Science, Khorramabad, Iran; ^5^ Student Research Committe, Lorestan University of Medical Science, Khorramabad, Iran; ^6^ Department of Cardiology, School of Medicine, Urmia University of Medical Sciences, Urmia, Iran

**Keywords:** Chronic Obstructive Pulmonary Disease, Microvascular Dysfunction, Ischemic Events, Stroke, Myocardial Infarction, Inflammation Markers

## Abstract

Chronic obstructive pulmonary disease (COPD), traditionally viewed as a localized pulmonary disorder, is now recognized as a systemic disease with far-reaching vascular consequences. Among its most concerning comorbidities are ischemic events such as myocardial infarction and stroke, which occur at disproportionately high rates in these patients, even after adjusting for shared risk factors like smoking. This unexpected persistence of risk has driven interest in the underlying mechanisms that might link lung dysfunction to vascular pathology. Emerging evidence suggests that microvascular dysfunction (MVD), characterized by endothelial injury, capillary rarefaction, and impaired vasoreactivity, may serve as a pivotal intermediary in this relationship. Systemic inflammation and oxidative stress, initiated in the lungs, appear to spill over into the circulation, damaging the vascular endothelium and setting the stage for atherosclerosis and ischemic injury. This review synthesizes current findings from molecular, imaging, and epidemiological studies to propose MVD as a central mechanistic bridge between COPD and ischemic events. We also examine therapeutic strategies that target endothelial health and highlight potential opportunities for early intervention. By reframing COPD as a disease with significant vascular implications, this review underscores the need for an integrated clinical approach that goes beyond pulmonary function and addresses systemic vascular health.

## Introduction

Chronic obstructive pulmonary disease (COPD) is a progressive and debilitating respiratory disorder that affects millions of people worldwide and ranks among the leading causes of global mortality [1] . Characterized by persistent airflow limitation and chronic airway inflammation, COPD has traditionally been viewed as a pulmonary disease with localized pathology. However, increasing evidence supports a broader systemic impact, with substantial extrapulmonary manifestations contributing to the overall disease burden [2]. Among these, car-diovascular and cerebrovascular diseases stand out as major comorbidities, with patients with COPD facing significantly increased risks of myocardial infarc-tion, stroke, and other ischemic events [3][4].

While cigarette smoking and aging are shared risk factors for both COPD and ischemic diseases, the incidence of vascular events in these patients remains ele-vated even after adjusting for these confounders [1]. This suggests that mecha-nisms intrinsic to COPD itself may play a pivotal role in the development of vascular pathology. In recent years, a growing body of literature has pointed to-ward microvascular dysfunction (MVD), a state of impaired endothelial func-tion, reduced vasodilatory capacity, and capillary rarefaction, as a key contribu-tor to cardiovascular risk in individuals with COPD [3][4].

Systemic inflammation and oxidative stress are hallmark features of COPD and are not confined to the lungs [3][5]. Pulmonary-derived inflammatory mediators, including interleukin-6 (IL-6), and tumor necrosis factor-alpha (TNF-α) can en-ter the circulation and exert deleterious effects on the vascular endothelium [3][4]. This "spillover" of inflammation from the pulmonary to systemic circula-tion is thought to promote endothelial activation, increase expression of adhe-sion molecules, reduce nitric oxide (NO) bioavailability, and ultimately lead to microvascular injury [4][5] . These processes set the stage for atherogenesis, plaque instability, and thrombotic events, linking the chronic inflammatory state of COPD to ischemic complications [3][4].

MVD has been well-documented in diseases such as diabetes and hypertension, but its role in COPD is only beginning to be elucidated [5]. Studies using ad-vanced vascular imaging, endothelial function testing, and biomarker analyses have shown early vascular impairment even in stable patients [3][4]. Further-more, acute exacerbations of COPD are associated with transient surges in sys-temic inflammation, during which the incidence of ischemic events significantly increases [6]. These findings highlight MVD not only as a comorbidity but as a potential pathophysiological bridge between pulmonary disease and vascular in-jury [3][4].

In this review, we examine the emerging role of MVD in COPD and its implica-tions for cardiovascular and cerebrovascular ischemic events. We explore the molecular and clinical evidence supporting this link, analyze potential diagnos-tic and therapeutic targets, and propose a conceptual framework in which COPD is redefined as a systemic inflammatory disorder with profound vascular conse-quences.

## Pathophysiology of COPD

COPD is primarily driven by chronic inflammation of the airways, lung paren-chyma, and pulmonary vasculature [7]. Its hallmark features include progressive airflow limitation, alveolar destruction, and mucus hypersecretion—most com-monly resulting from prolonged exposure to noxious stimuli such as cigarette smoke, air pollution, and occupational dusts [8]. These exposures initiate a cas-cade of immune responses that, over time, lead to irreversible structural changes and systemic consequences [9]. At the cellular level, the inhalation of irritants activates innate immune cells including alveolar macrophages, neutrophils, and dendritic cells [8]. These cells release proinflammatory mediators such as tumor necrosis factor-alpha (TNF-α), interleukin-6 (IL-6), and interleukin-1 beta (IL-1β), which recruit additional immune cells and amplify the inflammatory re-sponse [10].

CD8+ cytotoxic T cells and Th1/Th17 lymphocytes are also recruited and con-tribute to alveolar wall destruction and remodeling of the small airways [11].

One of the defining pathological features of COPD is airway remodeling, which involves goblet cell hyperplasia, smooth muscle hypertrophy, fibrosis, and nar-rowing of the bronchiolar lumen [8].

Simultaneously, emphysematous changes lead to the destruction of alveolar walls, loss of elastic recoil, and reduced surface area for gas exchange [7]. To-gether, these alterations cause airflow obstruction, hyperinflation, and impaired oxygenation [8]. Chronic inflammation in the pulmonary system can "spill over" into the systemic circulation, contributing to a low-grade systemic in-flammatory state [7].

Elevated levels of circulating cytokines, chemokines, and acute-phase proteins have been consistently observed in patients with both stable and exacerbated COPD [10]. This systemic inflammation plays a key role in mediating extrap-ulmonary effects, including skeletal muscle wasting, osteoporosis, metabolic syndrome, and endothelial dysfunction and vascular injury [12].

Another major contributor to COPD pathophysiology is oxidative stress [8]. Both exogenous sources (e.g., cigarette smoke) and endogenous sources (e.g., activated neutrophils) generate reactive oxygen species (ROS), which damage cellular structures, impair antioxidant defenses, and further exacerbate inflam-mation [7]. Oxidative stress also directly affects the endothelium, promoting vascular stiffness, reducing nitric oxide bioavailability, and facilitating micro-vascular dysfunction [9].

Additionally, hypoxia resulting from impaired ventilation-perfusion matching in COPD may further amplify systemic inflammation and oxidative injury [12]. Chronic hypoxia induces pulmonary vasoconstriction and vascular remodeling, which may contribute to pulmonary hypertension and right heart strain [8]. Be-yond the lungs, hypoxia may have deleterious effects on the brain, heart, and kidneys, especially in individuals with comorbid vascular disease [7].

Collectively, these mechanisms establish COPD not only as a pulmonary dis-ease but as a systemic inflammatory condition with clinically meaningful vascu-lar implications. [12].

The chronic inflammatory and oxidative environment in COPD creates fertile ground for microvascular dysfunction, making it a plausible pathophysiological link to ischemic cardiovascular and cerebrovascular events [8].

## Microvascular Dysfunction: Mechanisms and Evidence

MVD increasingly appears as a central mechanistic link between COPD and is-chemic cardiovascular outcomes [13][14]. Systemic inflammation in COPD con-tributes prominently to microvascular endothelial impairment. Chronic elevation of inflammatory cytokines, including IL-6 and TNF-α, promotes endothelial ac-tivation, leukocyte adhesion, and transmigration into the vessel wall, thereby initiating endothelial damage and dysfunction [13]. Additionally, neutrophils activated during persistent inflammation produce neutrophil extracellular traps (NETs), which directly damage endothelial cells and exacerbate microvascular injury [13][15].

Oxidative stress further amplifies endothelial dysfunction in COPD patients. Chronic exposure to reactive oxygen species (ROS), primarily generated from cigarette smoke and inflammatory cells, significantly reduces endothelial nitric oxide (NO) bioavailability [15]. The resultant NO deficiency impairs endotheli-um-dependent vasodilation, promoting vasoconstriction, thrombosis, and in-creased vascular permeability [16]. Furthermore, chronic hypoxia, a frequent complication in advanced COPD, induces endothelial remodeling and vasocon-striction through activation of hypoxia-inducible factors (HIFs) and upregulation of the renin-angiotensin system [14]. The combination of inflammation, oxida-tive stress, and hypoxia not only impairs endothelial function but also induces structural changes, such as vascular rarefaction and remodeling, exacerbating MVD [13].

Clinical and experimental evidence robustly supports the concept that MVD is prevalent and functionally significant in COPD patients. Clinical studies con-sistently report impaired endothelial-dependent dilation in these patients as measured by non-invasive assessments like flow-mediated dilation (FMD) and reactive hyperemia peripheral arterial tonometry (EndoPAT) [13][17]. For in-stance, patients with COPD exhibit substantially reduced coronary flow reserve (CFR) as assessed by cardiac magnetic resonance imaging (MRI), reflecting coronary MVD in the absence of large artery obstruction [14]. Similarly, com-puted tomography (CT) imaging of pulmonary vasculature frequently reveals "vascular pruning," characterized by diminished small pulmonary vessels, indic-ative of systemic microvascular impairment [18].

Emerging experimental data also link MVD directly to adverse cardiovascular outcomes in COPD patients. Studies consistently demonstrate increased cardio-vascular morbidity during acute COPD exacerbations, suggesting transient worsening of endothelial function and heightened thrombotic potential [19]. Population-based cohort studies confirm that COPD patients have a significantly elevated incidence of myocardial infarction, stroke, and peripheral vascular dis-ease, highlighting the systemic nature of MVD and its role in ischemic events [14][16]. Moreover, MVD observed via nailfold capillaroscopy and retinal imag-ing correlates with cardiovascular risk factors and adverse outcomes in COPD, underscoring the systemic impact of endothelial dysfunction beyond the pulmo-nary circulation [13].

## Ischemic Events in COPD Patients

COPD is well recognized as a systemic condition associated with elevated risk of atherosclerotic ischemic events, including myocardial infarction (MI), is-chemic stroke, and peripheral arterial disease (PAD) [16][20]. This heightened cardiovascular risk stems in part from shared risk factors such as cigarette smok-ing and older age, but COPD itself confers additional risk through disease-specific mechanisms. This disease is characterized by chronic systemic inflam-mation and intermittent hypoxemia, factors which can accelerate atherosclerosis, endothelial dysfunction, and thrombogenesis [21]. As a result, cardiovascular disease is a major cause of morbidity and mortality in COPD patients, account-ing for a substantial proportion of deaths in this population [21]. Epidemiologi-cal studies indicate that COPD roughly doubles the risk of acute MI in the gen-eral population, with about 10-17% of COPD patients experiencing an MI in their lifetime [20]. Even in individuals without prior cardiovascular disease, COPD has been linked to a higher incidence of coronary events - for example, a large cohort study showed that people with COPD had a 25% higher hazard of major adverse cardiovascular events (including MI, stroke, or cardiovascular death) compared to non-COPD counterparts after adjusting for traditional risk factors [22]. Mechanistic and clinical data also highlight acute COPD exacerba-tions as periods of particularly high cardiac risk; patients are significantly more likely to suffer an MI in the days following a severe exacerbation, underscoring the dynamic interplay between pulmonary inflammation and acute plaque rup-ture or thrombosis [23]. Importantly, when COPD patients do sustain an MI, they tend to have worse outcomes than those without COPD - studies report higher rates of acute complications (such as heart failure and arrhythmias) and increased mortality during follow-up in post-MI patients with COPD [21].

COPD is similarly associated with an increased risk of ischemic stroke in the general population. A recent systematic review and meta-analysis confirmed that this disease is an independent risk factor for stroke, reporting an overall odds ra-tio of approximately 1.4 for stroke occurrence in COPD patients compared to non-COPD individuals [24]. This cerebrovascular risk appears to be amplified during acute exacerbations of COPD; the meta-analysis noted that the risk of stroke was significantly elevated (approximately 1.5-fold higher) in the period surrounding COPD exacerbations [24]. The link between impaired lung function and stroke persists even after accounting for confounders like smoking and hy-pertension, suggesting that COPD-related pathophysiology contributes directly to cerebrovascular risk [13][24]. Moreover, outcomes after an ischemic stroke are worse in patients with COPD. Patients who suffer a stroke on a background of COPD have been shown to experience higher long-term mortality and poorer re-covery, likely reflecting both the added physiological strain of COPD and under-treatment or complexity of care in these patients [24]. This aligns with broader observations that coexistent COPD and cardiovascular disease lead to com-pounded morbidity - for instance, one large analysis found that the presence of COPD in stroke patients is associated with significantly increased risk of death in the years following the stroke, particularly in cases of ischemic stroke [13][24].

In addition to coronary and cerebrovascular events, COPD has been linked with an excess burden of peripheral arterial disease, another manifestation of systemic atherosclerosis. Population data demonstrate a markedly higher prevalence of PAD among individuals with COPD than in those without. For example, one cohort study reported PAD (defined by an abnormally low ankle-brachial index) in approximately 8-9% of COPD patients versus only around 2% in age- and sex-matched control subjects [20]. The prevalence of PAD in COPD increases further with greater airflow obstruction severity, indicating that more severe COPD confers greater peripheral vascular risk [20]. Mechanistically, the same chronic inflammatory and oxidative stress processes that affect the coronary and cerebral circulation in COPD likely also promote atherogenesis in peripheral ar-teries [25]. Clinically, the coexistence of COPD and PAD is important because it portends worse functional status and outcomes. COPD patients who develop PAD often have reduced exercise capacity and more pronounced mobility limi-tations than PAD patients without lung disease, partly due to the additive effects of claudication and dyspnea on exertion [20]. Furthermore, comorbid COPD ap-pears to worsen PAD prognosis: in a large vascular outcomes trial, PAD patients with COPD had higher rates of cardiovascular events (such as MI) during fol-low-up compared to PAD patients without COPD [26]. Other studies have noted that COPD patients with low ankle-brachial indices (indicative of PAD) face in-creased all-cause mortality over time, underscoring that peripheral ischemia in the context of COPD carries significant prognostic implications [20].

## MVDas a Pathophysiological Bridge

**Table:** Table1. Clinical and experimental markers of MVD in COPD

Marker Type	Specific Marker or Tool	Relevance in COPD	Relevance to MVD	Ref
	Pulmonary vascular pruning (CT)	Reflects loss of small pulmonary vessels	Indicates microvascular rarefaction	[18]
Imaging-based	Coronary Flow Reserve (CFR) via MRI or PET	Correlates with emphysema severity	Identifies coronary microvascular dysfunction	[14]
	Retinal vessel analysis	Non-invasive surrogate for systemic microvascular damage	Reflects systemic endothelial function	[13]
	Endothelial microparticles (EMPs)	High; marker of endothelial injury	Associated with vascular inflammation and dysfunction	[13]
Circulating biomarkers	CRP, IL-6, TNF-α	High; especially during exacerbations	Reflect systemic inflammation linked to MVD	[17]
	Asymmetric dimethylarginine (ADMA)	Impaired nitric oxide synthesis	Inhibits endothelial nitric oxide synthase	[16]
Functional vascular tests	Flow-mediated dilation (FMD)	Low	Directly measures endothelial-dependent vasodilation	[13]
	Reactive hyperemia index (EndoPAT)	Abnormal in COPD with comorbidities	Non-invasive test of systemic endothelial dysfunction	[13]
Inflammatory/ oxidative	Oxidized LDL, isoprostanes	Elevated oxidative stress	Linked with endothelial damage and atherosclerosis	[15]
Metabolic markers	Microalbuminuria	Observed in some Sudies	Reflects microvascular leakage and endothelial dysfunction	[16]

Emerging evidence underscores MVD as a critical pathophysiological link be-tween COPD and ischemic cardiovascular events. This connection is driven by shared mechanisms, including endothelial dysfunction, systemic inflammation, oxidative stress, and hypoxia-induced vascular remodeling, which collectively impair microvascular homeostasis and predispose individuals to ischemic out-comes [3][5].

### Endothelial Dysfunction and Impaired Vasoreactivity

The coronary and systemic microvasculature in COPD patients exhibit endothe-lial dysfunction, characterized by reduced nitric oxide (NO) bioavailability and impaired endothelium-dependent vasodilation. This is exacerbated by chronic hypoxia, a hallmark of COPD, which disrupts mitochondrial respiration and amplifies oxidative stress, further damaging endothelial cells [5][27]. Studies us-ing invasive coronary function testing have demonstrated that COPD patients frequently exhibit diminished coronary flow reserve (CFR < 2.5) and elevated microvascular resistance (IMR ≥ 25), reflecting impaired vasodilatory capacity and structural microvascular remodeling [28][29]. These abnormalities correlate with increased susceptibility to myocardial ischemia, even in the absence of ob-structive coronary artery disease [3][30].

### Systemic Inflammation and Oxidative Stress

COPD-associated systemic inflammation, marked by elevated cytokines (e.g., IL-6, TNF-α) and reactive oxygen species (ROS), directly contributes to micro-vascular damage (Figure-1) [31][32]. Pro-inflammatory mediators promote endo-thelial apoptosis and enhance vascular permeability, while ROS reduce NO syn-thase activity, fostering a pro-constrictive vascular milieu [27][30]. This inflam-matory cascade is compounded by COPD-related hypoxia, which activates hy-poxia-inducible factor-1α (HIF-1α), driving oxidative stress and capillary rare-faction a structural hallmark of MVD [5][27]. Notably, these pathways are also implicated in atherosclerosis progression, creating a bidirectional relationship between COPD and ischemic events [3][4]. Table 1 demonstrated common clini-cal and experimental markers.

### Autonomic Dysregulation and Hypoxia

Chronic hypoxia in COPD disrupts autonomic balance, increasing sympathetic tone and reducing parasympathetic activity. This imbalance exacerbates MVDby promoting vasoconstriction and endothelial injury through α-adrenergic receptor activation [27]. Preclinical models further suggest that intermittent hypoxia, common in COPD exacerbations, induces microvascular endothelial glycocalyx shedding, impairing barrier function and promoting leukocyte adhesion a pre-cursor to thrombotic events [5][27].

### Clinical and Prognostic Implications

Recent cohort studies highlight that COPD patients with MVD face a 2-3-fold higher risk of major adverse cardiovascular events (MACE), including MI and heart failure, compared to those without microvascular impairment [3][29]. Non-invasive imaging modalities, such as positron emission tomography (PET) and cardiac magnetic resonance (CMR), have validated these associations by reveal-ing reduced myocardial perfusion reserve (MPR < 1.5) and subendocardial is-chemia in COPD cohorts [4][29]. Furthermore, MVD in COPD correlates with worse pulmonary function (e.g., FEV1 decline) and higher mortality, suggesting a synergistic pathophysiological interplay [27].

**Figure-2:**
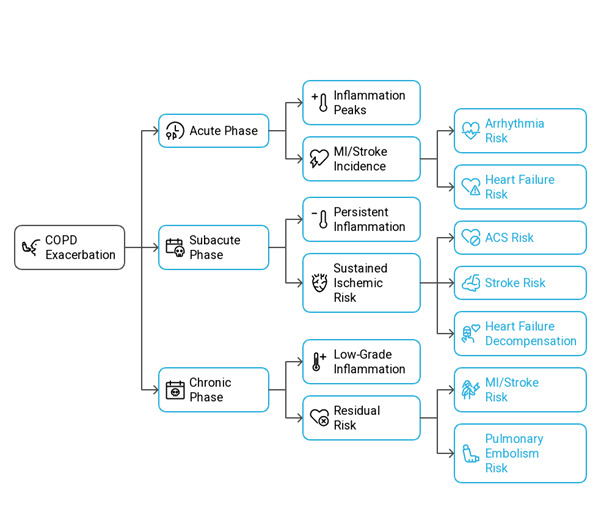
Figure 2. Temporal Association Between COPD Exacerbations and Ischemic Risk. [31, 32]

### Therapeutic Opportunities

Targeting MVD in COPD requires a multifaceted approach. Pharmacological strategies, such as angiotensin-converting enzyme inhibitors (ACEIs) and statins, improve endothelial function by enhancing NO bioavailability and re-ducing oxidative stress [27][28]. Preclinical data also support the use of SGLT2 inhibitors, which attenuate hypoxia-induced endothelial inflammation and fibro-sis [27][33] . Non-pharmacological interventions, including pulmonary rehabili-tation and oxygen therapy, may mitigate hypoxia-driven microvascular damage, though further clinical trials are needed to validate these benefits [33].

## Therapeutic Implications

Emerging insights into MVD as a bridge between COPD and ischemic events have highlighted novel therapeutic strategies targeting shared pathophysiologi-cal pathways, including endothelial dysfunction, systemic inflammation, oxida-tive stress, and hypoxia-induced vascular remodeling [34]. These approaches aim to mitigate cardiovascular risk in COPD patients while addressing pulmo-nary limitations, though challenges remain in translating mechanistic insights into clinical practice [35].

## Pharmacological Interventions

**Table:** Table2. Therapeutics with dual benefit on COPD and MVD

**Therapeutic Class **	**Drug Example(s)**	**Benefit in COPD**	**Benefit in Microvascular Dysfunction **	**References**
**Statins**	Simvastatin, Atorvastatin	Anti-inflammatory, reduces exacerbations	Improves endothelial function, reduces oxidative stress, enhances nitric oxide bioavailability	[15][42][43]
**ACE inhibitors**	Ramipril, Enalapril	May reduce pulmonary hypertension	Improves endothelial function, reduces vascular remodeling	[13][16]
**Angiotensin Receptor Blockers (ARBs)**	Losartan, Telmisartan	Reduces emphysema progression	Reduces systemic inflammation, prevents endothelial dysfunction	[14][15]
**Phosphodiesterase-5 inhibitors**	Sildenafil, Tadalafil	Reduces pulmonary artery pressure, improves exercise capacity	Enhances endothelial function, promotes vasodilation	[13][15]
**Beta-blockers**	Bisoprolol, Nebivolol	May reduce cardiovascular mortality (careful selection required)	Improves endothelial function, reduces arterial stiffness (selected agents)	[19][43]
**Antioxidants**	N-acetylcysteine (NAC)	Reduces exacerbations, mucus viscosity	Reduces oxidative stress, protects endothelial cells	[13][15]
**Anti-inflammatory agents**	Roflumilast	Reduces exacerbation frequency	Decreases systemic inflammation, may indirectly benefit endothelial function	[15][16]

Table 2 shows common medication with dual benefit on COPD and MVD. An-giotensin-converting enzyme inhibitors (ACEIs) and angiotensin receptor block-ers (ARBs) improve endothelial function by enhancing nitric oxide (NO) bioa-vailability and reducing oxidative stress, thereby alleviating microvascular im-pairment in COPD patients [36]. Statins, beyond lipid-lowering effects, reduce systemic inflammation and stabilize endothelial cells, potentially lowering the risk of ischemic events in COPD cohorts with MVD [37]. Sodium-glucose co-transporter-2 (SGLT2) inhibitors, originally developed for diabetes, have shown promise in preclinical models by attenuating hypoxia-induced endothelial in-flammation and fibrosis, offering dual cardiopulmonary benefits [38]. Endo-thelin receptor antagonists, such as Zibotentan, are under investigation for their ability to counteract vasoconstrictive pathways exacerbated by chronic hypoxia, with ongoing trials like PRIZE exploring their efficacy in MVD-associated an-gina [39].

### Non-Pharmacological and Adjunctive Strategies

Pulmonary rehabilitation programs, combining exercise training and respiratory therapy, enhance endothelial function and reduce systemic inflammation, indi-rectly improving microvascular health in COPD patients [40]. Long-term oxy-gen therapy (LTOT) may mitigate hypoxia-driven microvascular remodeling, though evidence remains mixed, necessitating further trials to optimize dosing and patient selection [33]. Smoking cessation remains a cornerstone interven-tion, as smoking perpetuates endothelial dysfunction and oxidative stress, accel-erating MVD progression [41]. Emerging therapies, such as autologous CD34+ cell transplantation, aim to regenerate damaged microvasculature, with early-phase trials demonstrating improved myocardial perfusion in ischemic syn-dromes [35].

### Personalized Medicine and Future Directions

Stratified approaches based on endotype-specific biomarkers (e.g., endothelin-1, IL-6, and CRP) may optimize treatment efficacy by targeting dominant patho-logical mechanisms in individual patients [40]. For instance, patients with auto-nomic dysregulation may benefit from beta-blockers or neuromodulation thera-pies, while those with chronic inflammation could respond better to IL-6 or TNF-α inhibitors[41]. Large-scale trials like WARRIOR are evaluating intensive medical therapy (statins, ACEIs, and aspirin) in women with ischemia and non-obstructive coronary arteries (INOCA), a population overlapping with COPD-related MVD [38]. Additionally, advancements in non-invasive imaging (e.g., PET and CMR) enable dynamic monitoring of microvascular perfusion, guiding therapeutic adjustments and risk stratification [36].

## Knowledge Gaps and Future Directions

### Diagnostic Limitations in Identifying Microvascular Dysfunction

Despite clear associations between COPD and MVD, significant challenges per-sist in clinically diagnosing MVD. Currently, available diagnostic methods such as coronary reactivity tests, positron emission tomography (PET), and cardiac magnetic resonance imaging (MRI) are invasive, expensive, and rarely applied in routine COPD management [14]. Reliable non-invasive biomarkers that iden-tify microvascular impairment specifically in COPD patients remain unavaila-ble, as current markers like C-reactive protein (CRP) and homocysteine are non-specific and demonstrate inconsistent predictive value [17]. Recent research suggests promising alternatives, including the quantification of endothelial mi-croparticles or endothelial cell-derived circulating factors, yet these approaches require further validation [13]. Advanced imaging techniques, such as computed tomography (CT)-based vascular analysis, have identified COPD-associated vascular changes like vascular pruning; however, these methods remain confined primarily to research settings and are not broadly accessible for clinical use [18]. Future diagnostic advances could focus on developing multimodal approaches combining biomarkers and imaging to enable early detection of MVD, thus fa-cilitating timely interventions and improved patient outcomes.

### Treatment Challenges for MVDin COPD

The absence of targeted treatments specifically addressing MVD in COPD rep-resents a critical therapeutic gap. While COPD treatments like bronchodilators and inhaled corticosteroids target respiratory symptoms and airflow limitation, they do not specifically address underlying vascular abnormalities [16]. Cardio-vascular agents, such as statins, angiotensin receptor blockers (ARBs), and ACE inhibitors, have shown preclinical promise in improving endothelial function and reducing pulmonary and systemic inflammation; however, clinical trials in COPD populations have yielded mixed or negative results [42]. Notably, trials investigating beta-blocker therapy (metoprolol), were prematurely terminated due to increased adverse outcomes, emphasizing the complexity of therapeutic management in COPD patients with cardiovascular comorbidities [43]. These findings highlight the necessity for targeted pharmacological therapies explicitly designed to protect or restore endothelial function in COPD. Novel strategies, including endothelial progenitor cell therapies, antioxidant agents, and anti-inflammatory drugs specifically targeting vascular pathways, should be explored in future research [15].

### Opportunities for Clinical Innovation

Innovative clinical trial designs and precision medicine approaches present sub-stantial opportunities to address current knowledge gaps in the management of MVD in COPD. Traditional clinical trials have separately focused on pulmonary outcomes or cardiovascular endpoints, rarely integrating these aspects despite their interconnection [16]. Future trials should prioritize combined endpoints such as measures of endothelial function, vascular biomarkers, and cardiovascu-lar events in addition to traditional respiratory metrics. Moreover, timing inter-ventions to periods of heightened cardiovascular risk, such as immediately after COPD exacerbations, may enhance the efficacy of targeted vascular therapies [19]. Precision medicine represents another key direction, emphasizing patient stratification according to specific COPD phenotypes characterized by distinct vascular pathologies [14]. For instance, advanced imaging and molecular profil-ing could identify subsets of patients with greater microvascular involvement, enabling targeted, individualized interventions. This personalized strategy could significantly improve the effectiveness of interventions by directing therapies to patients most likely to benefit, ultimately bridging the gap between pulmonary and cardiovascular management in COPD [15][19].

## Conclusion

COPD is no longer merely a pulmonary ailment, it is a systemic disorder with profound cardiovascular and cerebrovascular implications. This review has traced the intricate pathophysiological pathway through which MVD emerges as a central mechanistic bridge linking COPD to a spectrum of ischemic events, in-cluding myocardial infarction, stroke, and peripheral arterial disease. The con-vergence of systemic inflammation, oxidative stress, chronic hypoxia, and auto-nomic imbalance in COPD patients results in widespread endothelial injury, im-paired vasoreactivity, and vascular remodeling—hallmarks of MVD that directly elevate cardiovascular risk.

Clinical and imaging evidence increasingly supports the presence of MVD in COPD populations, even among those without traditional cardiovascular risk factors. Moreover, acute exacerbations of COPD function as high-risk periods for vascular events, underscoring the dynamic and bidirectional relationship be-tween pulmonary inflammation and systemic vascular injury. Despite these in-sights, diagnostic and therapeutic approaches remain fragmented, with current interventions often failing to address the vascular dimensions of COPD.

To fully realize the benefits of this emerging knowledge, there is a pressing need for integrated clinical strategies that incorporate vascular assessment into COPD management. Future research should focus on developing specific diagnostic bi-omarkers for MVD, refining non-invasive imaging modalities, and testing endo-thelium-targeted therapies in well-designed, stratified clinical trials. By refram-ing COPD as a systemic inflammatory syndrome with vascular consequences, clinicians and researchers alike can move toward a more holistic, multidiscipli-nary approach ultimately reducing both pulmonary and vascular morbidity and mortality in this high-risk population.

## Conflict of Interest

The authors declare no conflicts of interest.

## References

[R1] Chen H, Luo X, Du Y, He C, Lu Y, Shi Z, et al (2023). Association between chronic obstructive pulmonary disease and cardiovascular disease in adults aged 40 years and above: data from NHANES 2013–2018. BMC Pulm Med.

[R2] Safiri S, Carson-Chahhoud K, Noori M, Nejadghaderi SA, Sullman MJ, Heris JA, et al (2022). Burden of chronic obstructive pulmonary disease and its attributable risk factors in 204 countries and territories, 1990-2019: results from the Global Burden of Disease Study 2019. BMJ.

[R3] Gurgoglione FL, Benatti G, Denegri A, Donelli D, Covani M, De Gregorio M, et al (2025). Coronary Microvascular Dysfunction: Insights on Prognosis and Future Perspectives. Rev Cardiovasc Med.

[R4] Montone RA, et al (2023). Coronary microvascular dysfunction: A review of recent progress and clinical implications. Front Cardiovasc Med.

[R5] Vancheri F, Longo G, Vancheri S, Henein M (2020). Coronary Microvascular Dysfunction. J Clin Med.

[R6] Pirera E, Di Raimondo D, D'Anna L, Tuttolomondo A (2025). Risk trajectory of cardiovascular events after an exacerbation of chronic obstructive pulmonary disease: A systematic review and meta-analysis. Eur J Intern Med.

[R7] Corhay JL, Bonhomme O, Heinen V, Moermans C, Louis R (2022). Chronic obstructive pulmonary disease A chronic inflammatory disease. Rev Med Liege.

[R8] Barnes PJ, Shapiro SD, Pauwels RA (2003). Chronic obstructive pulmonary disease: molecular and cellular mechanisms. Eur Respir J.

[R9] Angelis N, Porpodis K, Zarogoulidis P, Spyratos D, Kioumis I, Papaiwannou A, et al (2014). Airway inflammation in chronic obstructive pulmonary disease. J Thorac Dis.

[R10] Sohal SS, Eapen MS, Shukla SD, Courtney JM, Mahmood MQ, Walters EH (2014). Novel insights into chronic obstructive pulmonary disease (COPD): an overview. EMJ Respir.

[R11] Matanić D, Flego V, Barković I, Zeba I, Kupanovac Ž, Bulat-Kardum L (2009). Chronic obstructive pulmonary disease multisistemic disease. Medicina.

[R12] Mehta AA, Jose RJ, Page C, Barnes PJ (2022). Microvascular endothelial dysfunction in COPD: From pathophysiology to therapeutic targets. Eur Respir Rev.

[R13] Murashima M, Nishida M, Kataoka Y, Fukuda D, Shimada K, Shintani Y (2024). Coronary microvascular dysfunction: Linking chronic obstructive pulmonary disease and ischemic heart disease. J Cardiol.

[R14] Barnes PJ (2021). Oxidative stress-based therapeutics in COPD. Redox Biol.

[R15] Balbirsingh V, Mohammed AS, Glaser J, Zein JG (2022). Cardiovascular Disease in Chronic Obstructive Pulmonary Disease: A Comprehensive Review. Curr Opin Pulm Med.

[R16] Preston IR, Hill NS, Gambardella LS, Warburton RR, Klinger JR (2020). Circulating biomarkers of endothelial dysfunction in pulmonary hypertension and chronic obstructive pulmonary disease. Chest.

[R17] Labaki WW, Gu T, Murray S, Curtis JL, Martinez CH, Han MK, et al (2020). Vascular pruning on computed tomography and risk of mortality in COPD. Am J Respir Crit Care Med.

[R18] Kunisaki KM, Dransfield MT (2022). Cardiovascular risk and COPD: The devil is in the details. Chest.

[R19] Papaporfyriou A, Loutradis C, Tzatzagou G, Bakogiannis C (2023). Chronic Obstructive Pulmonary Disease and Cardiovascular Disease: An Update. Eur J Clin Invest.

[R20] Bax S, Medina-Gomez C, Koudstaal PJ, Ikram MA, Brusselle GG, Lahousse L (2020). Chronic Obstructive Pulmonary Disease and the Risk of Cardiovascular Disease: A Population-Based Cohort Study. Eur Respir J.

[R21] Maclagan LC, Gershon AS, Tanuseputro P, Austin PC, Beach J, Kendzerska T (2023). Risk of Cardiovascular Events in Patients with COPD Compared to Those Without COPD. Chest.

[R22] Yang J, Luo H, Wang Y, Chen R (2024). Association Between Acute Exacerbation of COPD and Risk of Myocardial Infarction: A Systematic Review and Meta-analysis. Respir Res.

[R23] Ding W, Huang X, Wang M, Liang C (2023). Chronic Obstructive Pulmonary Disease and Risk of Stroke: A Systematic Review and Meta-Analysis. Front Neurol.

[R24] Brassington K, Selemidis S, Bozinovski S, Vlahos R (2019). New Frontiers in the Treatment of Comorbid Cardiovascular Disease and Chronic Obstructive Pulmonary Disease. Clin Sci.

[R25] Galani IE, Chatzimichail EA, Xanthopoulos A, Aggeli C, Siogas K, Karatzis E (2021). Chronic Obstructive Pulmonary Disease is Associated With Increased Cardiovascular Events in Peripheral Artery Disease Patients. Angiology.

[R26] La Vecchia G, Fumarulo I, Caffè A, Chiatto M, Montone RA, Aspromonte N (2024). Microvascular Dysfunction across the Spectrum of Heart Failure Pathology: Pathophysiology, Clinical Features and Therapeutic Implications. Int J Mol Sci.

[R27] Xu J, Lo S, Juergens CP, Leung DY (2021). Impact of Targeted Therapies for Coronary Microvascular Dysfunction as Assessed by the Index of Microcirculatory Resistance. J Cardiovasc Transl Res.

[R28] Zimmerli A, Salihu A, Antiochos P, Lu H, Pitta Gros B, Berger A, et al (2025). Evolution of Coronary Microvascular Dysfunction Prevalence over Time and Across Diagnostic Modalities in Patients with ANOCA: A Systematic Review. J Clin Med.

[R29] Abramik J, Mariathas M, Felekos I (2025). Coronary Microvascular Dysfunction and Vasospastic Angina—Pathophysiology, Diagnosis and Management Strategies. J Clin Med.

[R30] Graul EL, Nordon C, Rhodes K, Marshall J, Menon S, Kallis C, et al (2024). Temporal Risk of Nonfatal Cardiovascular Events After Chronic Obstructive Pulmonary Disease Exacerbation: A Population-based Study. Am J Respir Crit Care Med.

[R31] Wallström O, Stridsman C, Lindberg A, Nyberg F, Vanfleteren LEGW (2024). Exacerbation History and Risk of Myocardial Infarction and Pulmonary Embolism in COPD. Chest.

[R32] Siafakas NM, Antoniou KM, Tzortzaki EG (2007). Role of angiogenesis and vascular remodeling in chronic obstructive pulmonary disease. Int J Chron Obstruct Pulmon Dis.

[R33] Rehan R, Yong A, Ng M, Weaver J, Puranik R (2023). Coronary microvascular dysfunction: a review of recent progress and clinical implications. Front Cardiovasc Med.

[R34] Bhogal S, Batta A, Mohan B (2025). Invasive assessment of coronary microvascular disease and its implications. World J Cardiol.

[R35] Scarica V, Rinaldi R, Animati FM, Manzato M, Montone RA (2025). Coronary microvascular dysfunction: pathophysiology, diagnosis, and therapeutic strategies across cardiovascular diseases. EXCLI J.

[R36] Potere N, Bonaventura A, Abbate A (2024). Novel therapeutics and upcoming clinical trials targeting inflammation in cardiovascular diseases. Arterioscler Thromb Vasc Biol.

[R37] Pepine CJ (2022). Treatment options for coronary microvascular dysfunction. J Am Coll Cardiol.

[R38] Crea F, Montone RA, Rinaldi R (2022). Pathophysiology of coronary microvascular dysfunction. Circ J.

[R39] Taqueti VR, Di Carli MF (2023). Circulating biomarkers in coronary microvascular dysfunction. Biomolecules.

[R40] Johnson PL, Shekhar A (2012). An animal model of panic vulnerability with chronic disinhibition of the dorsomedial/perifornical hypothalamus. Physiol Behav.

[R41] Criner GJ, Connett JE, Aaron SD, Albert RK, Bailey WC, Casaburi R, et al (2019). Simvastatin for the prevention of exacerbations in moderate-to-severe COPD. N Engl J Med.

[R42] Dransfield MT, Voelker H, Bhatt SP, Brenner K, Casaburi R, Come CE, et al (2019). Metoprolol for the prevention of acute exacerbations of COPD. N Engl J Med.

